# UHRF1 Suppresses HIV-1 Transcription and Promotes HIV-1 Latency by Competing with p-TEFb for Ubiquitination-Proteasomal Degradation of Tat

**DOI:** 10.1128/mBio.01625-21

**Published:** 2021-08-31

**Authors:** Taizhen Liang, Qiao Zhang, Ziyao Wu, Pei Chen, Yifan Huang, Shuwen Liu, Lin Li

**Affiliations:** a Guangdong Provincial Key Laboratory of New Drug Screening, Guangzhou Key Laboratory of Drug Research for Emerging Virus Prevention and Treatment, School of Pharmaceutical Sciences, Southern Medical University, Guangzhou, China; b School of Medicine, Jinan University, Guangzhou, China; McMaster University

**Keywords:** HIV-1 latency, UHRF1, Tat, p-TEFb, RING finger

## Abstract

HIV-1 remains incurable due to viral reservoirs, which lead to durably latent HIV infection. Identifying novel host factors and deciphering the molecular mechanisms involved in the establishment and maintenance of latency are critical to discover new targets for the development of novel anti-HIV agents. Here, we show that ubiquitin-like with PHD and RING finger domain 1 (UHRF1) modulates HIV-1 5′-long terminal repeat (LTR)-driven transcription of the viral genome as a novel HIV-1 restriction factor. Correspondingly, UHRF1 depletion reversed the latency of HIV-1 proviruses. Mechanistically, UHRF1 competed with positive transcription factor b (p-TEFb) for the binding to the cysteine-rich motifs of HIV-1 Tat via its TTD, PHD, and RING finger domains. Furthermore, UHRF1 mediated K48-linked ubiquitination and proteasomal degradation of Tat in RING-dependent ways, leading to the disruption of Tat/cyclin T1/CDK9 complex and consequential impediment of transcription elongation. In summary, our findings revealed that UHRF1 is an important mediator of HIV-1 latency by controlling Tat-mediated transcriptional activation, providing novel insights on host-pathogen interaction for modulating HIV-1 latency, beneficial for the development of anti-AIDS therapies.

## INTRODUCTION

Latent HIV-1 infection in resting CD4^+^ T lymphocytes is the major obstacle to complete eradication of the virus in patients after suppressive combination antiretroviral therapy (cART) (1, 2). Therefore, lifelong cART is required. If interrupted, viremia will rapidly rebound. To eliminate the HIV-1 latent reservoir, it is extremely important to understand the molecular mechanisms involved in controlling the stability of HIV-1 latency, which would instruct us to design effective strategies for destabilizing latency and the elimination of latent reservoirs. The host machinery plays a critical role in the maintenance of HIV-1 latency via silencing the expression of HIV-1 genes. The 5′-long terminal repeat (LTR) of integrated HIV-1 proviruses containing promoter/enhancer elements determines the outcome of HIV-1 gene expression and the switch between the lytic/latent stages and is subjected to substantial regulation by the host machinery. Over recent decades, the accumulated evidence shows that a panel of host factors is involved in regulation of HIV-1 latency. Genome-wide CRISPR-Cas9 screens identified FTSJ3, TMEM178A, NICN1, and the integrator complex as host regulators that promote HIV-1 latency (3), and a total of 471 genes were demonstrated to play key roles in HIV-1 initiation or the enforcement of latency through transposon-accessible chromatin sequencing (ATAC-seq) (4). Recently, our group identified the host factor heat shock factor 1 (HSF1) as a universal factor in latent HIV-1 reactivation induced by latency-reversing agents (LRAs) that is indispensable in HIV-1 transcription (5).

The Tat protein encoded by the HIV-1 genome is a potent activator of the viral LTR promoter required for viral replication. Tat recruits cellular positive transcriptional elongation factor b (p-TEFb) to the transcription response element (TAR), resulting in the phosphorylation of negative elongation factor (NELF), DRB sensitivity-inducing factor (DSIF), and C-terminal domain (CTD) of RNA polymerase II (RNA Pol II), followed by the release of the stalled polymerase, and intensely promotes the transcription elongation of HIV-1 (6). Insufficient Tat expression can lead to viral silencing and latency establishment (7). Recent studies show that HIV-1 Tat is subjected to strict regulation by various mechanisms involving autophagy, lysosome (8), or proteasome-mediated protein degradation (9). Ubiquitination plays a significant role in modulating host-pathogen interactions critical for HIV-1 pathogenesis. The lncRNA NRON promotes Tat degradation through ubiquitin proteasome systems (10). Similarly, the proto-oncoprotein Hdm2 interacts with Tat and facilitates its ubiquitination *in vitro* and *in vivo* (11). Tat associates with multiple host factors to hijack host machinery, which benefits HIV-1 propagation and/or causes HIV-1 pathogenesis (12–14). Thus, exploring novel host factors associated with Tat and revealing their role in HIV-1 latency may provide novel insights into the molecular basis underlying the establishment and maintenance of HIV-1 latency.

Ubiquitin-like PHD and RING finger domain-containing protein 1 (UHRF1), also known as nuclear protein 95 (Np95) in mice or inverted CCAAT box-binding protein of 90 kDa (ICBP90) in humans, is a unique epigenetic effector important for multiple aspects of epigenetic regulation, including the recognition and maintenance of various histone modifications (15, 16). Early studies show the canonical function of UHRF1 as a multifunctional protein is in the recruitment of DNA methyltransferase 1 (DNMT1) to DNA loci, thereby facilitating DNA hypermethylation (15, 17), which plays important roles in cell proliferation, cell cycle progression, and tumor aggressiveness (16, 18, 19). In addition, the structural basis shows UHRF1 acts as a transcriptional repressor through its binding to unmodified Arg-2 and trimethylated Lys-9 of histone H3 by the PHD and TTD domains, respectively (20). In terms of its relevance to the study of HIV-1, UHRF1 has been found to be associated with HIV-1 transcription (21), but the role of UHRF1 in regulating HIV-1 latency remains elusive and requires further investigation.

In this study, we investigated the potential role of UHRF1 in HIV-1 replication and the reactivation of latent HIV-1. Our results showed that UHRF1 acts as a positive modulator of viral latency and hinders HIV-1 gene expression. Mechanistically, UHRF1 competes with p-TEFb for the association with HIV-1 Tat, promotes the proteasomal degradation of Tat via K48-linked ubiquitination, and further interferes with transcriptional elongation of HIV-1 in cellular models of latency, leading to HIV-1 transcription repression and viral latency.

## RESULTS

### UHRF1 potently inhibits HIV-1 replication.

**FIG 1:**
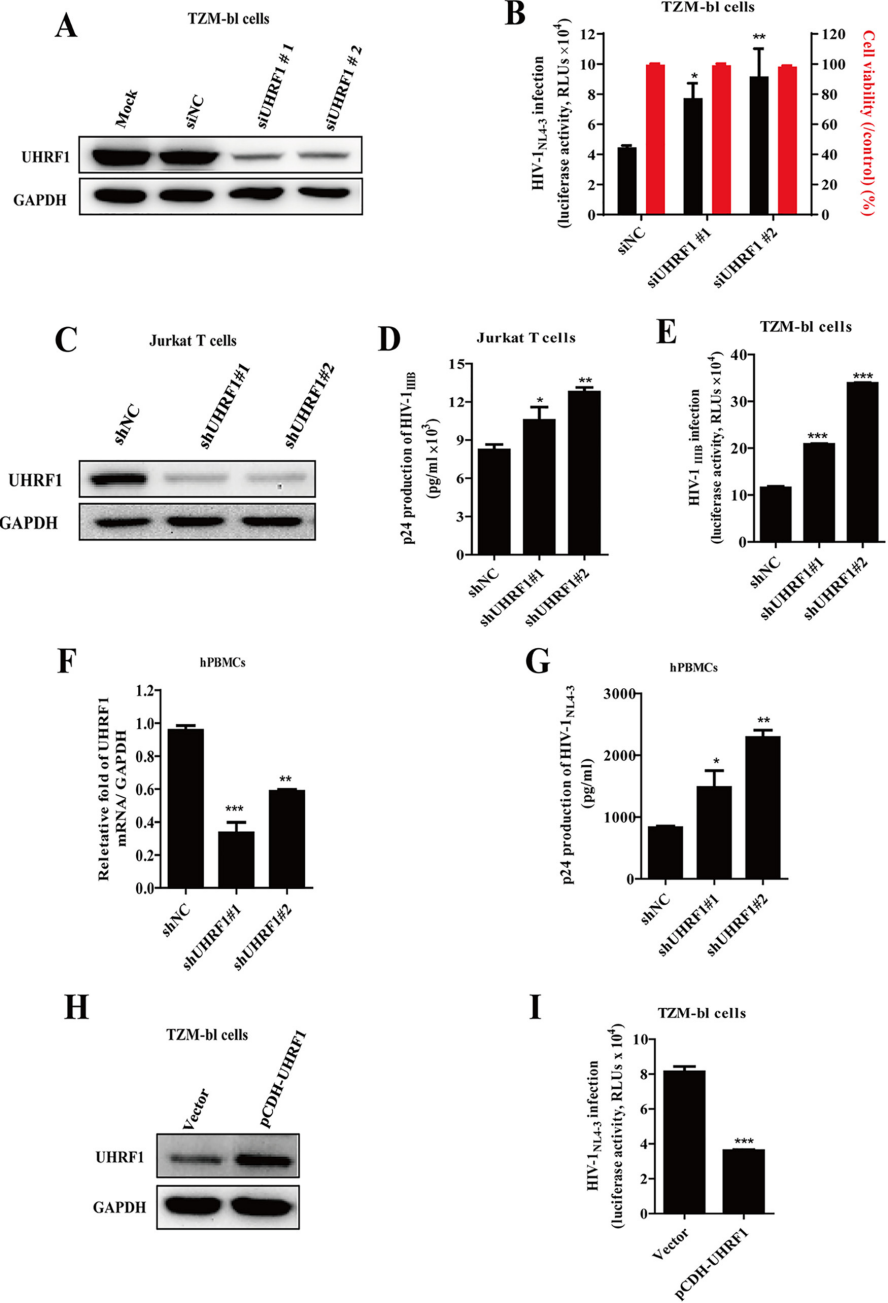
UHRF1 inhibits HIV-1 replication. (A) Immunoblot analysis of the efficiency of endogenous UHRF1 knockdown by the indicated siRNA in TZM-bl cells. (B) The cells were infected with replication-competent HIV-1_NL4-3_ and monitored for viral replication by luciferase activity (black bar) and cell viability (red bar) by flow cytometry. (C) Jurkat T cells were stably transduced with UHRF1 shRNA and UHRF1 expression was detected by immunoblotting. (D and E) Cells were infected with HIV-1_IIIB_, and cell culture supernatants were harvested for detection by Gag p24 capture ELISA (D) or detection of HIV-1 production in TZM-bl indicator cells (E). (F) RT-qPCR analysis of the efficiency of endogenous UHRF1 knockdown in PBMCs. (G) Cells were infected with HIV-1_NL4-3_ and monitored for viral replication by Gag p24 capture ELISA. (H and I) UHRF1 overexpression levels were detected by immunoblotting (H), and HIV-1 infection was detected by measuring luciferase activity (I). Data are presented as the means ± standard deviations (SD) from three independent experiments. Statistical significance was determined by unpaired Student's *t* test or one-way ANOVA followed by Dunnett’s multiple-comparison *post hoc* test: *, *P < *0.05; **, *P < *0.01; ***, *P < *0.001.

To investigate the role of UHRF1 in the regulation of HIV-1 replication, we first analyzed whether the depletion of UHRF1 would affect cell infection with the replication-competent HIV-1_NL4-3_ virus. Here, the TZM-bl cell line, integrated with HIV-1 LTR-driven luciferase, is sensitive to the LTR-mediated reactivation of latent HIV-1 (22). Endogenous UHRF1 was successfully depleted in TZM-bl cells using two different small interfering RNAs (siRNAs) (siUHRF1#1 and #2) (Fig. 1A), and the cells were then infected with HIV-1_NL4-3_ for 24 h. Compared with siNC treatment, the siUHRF1 group modestly increased luciferase activity up to 1.5- to 2-fold, suggesting that UHRF1 depletion facilitates LTR-mediated HIV-1 replication (Fig. 1B). No effect on cell viability was observed in response to UHRF1 silencing (Fig. 1B). To further explore the function of UHRF1 in HIV-1 replication, stable Jurkat T cells with UHRF1 knockdown generated by UHRF1-specific short hairpin RNA (shRNA) (shUHRF1#1 and #2) lentiviruses (Fig. 1C) were infected with HIV-1_IIIB_ for 24 h. Consistent with luciferase activity results, downregulation of UHRF1 expression modestly increased p24 production of HIV-1_IIIB_ released into the supernatant in Jurkat T cells, as quantified by enzyme-linked immunosorbent assay (ELISA) (Fig. 1D). The released virion particles in the cell supernatant were incubated with TZM-bl cells for another 48 h. As shown in Fig. 1E, UHRF1 depletion promoted the release of virion particles into the cell supernatant, as the luciferase activity was modestly increased up to 1.8- to 2.9-fold in TZM-bl cells. Correspondingly, human peripheral blood mononuclear cells (PBMCs) isolated from the blood of healthy donors at Nanfang Hospital were transduced with lentiviruses encoding UHRF1-specific shRNA for UHRF1 knockdown (Fig. 1F). After the treatment with replication-competent HIV-1_NL4-3_, UHRF1 knockdown increased HIV-1 replication, as demonstrated by increased viral particle synthesis and release quantified by ELISA (Fig. 1G). Simultaneously, we also observed obvious increases in viral Gag and LTR mRNA expression in Jurkat T cells with UHRF1 knockdown (see Fig. S1 in the supplemental material).

10.1128/mBio.01625-21.1 FIG S1 UHRF1 potently inhibits HIV-1 replication. The mRNA levels of HIV-1 Gag and LTR in Jurkat T cells infected with HIV-1_IIIB_ with UHRF1 knockdown were detected by RT-qPCR. Data are presented as the mean ± SD from three independent experiments. *, *P < *0.05; ***, *P < *0.01 (unpaired Student’s *t* test). Download FIG S1, TIF file, 0.6 MB. Copyright © 2021 Liang et al. 2021 Liang et al. https://creativecommons.org/licenses/by/4.0/ This content is distributed under the terms of the Creative Commons Attribution 4.0 International license.

Since the loss of UHRF1 facilitated HIV-1 replication, we asked whether UHRF1 overexpression would suppress HIV-1 replication. UHRF1 was transiently overexpressed in TZM-bl cells (Fig. 1H), and then cells were infected with HIV-1_NL4-3_. Correlating with previous results, luciferase activity was reduced when overexpressing UHRF1 in TZM-bl cells compared with the vector group (Fig. 1I). Taken together, these results suggest that UHRF1 inhibits HIV-1 replication and may be an HIV-1 restriction factor for HIV-1 infection and replication.

### UHRF1 associates with HIV-1 5′-LTR and antagonizes viral transcription.

**FIG 2:**
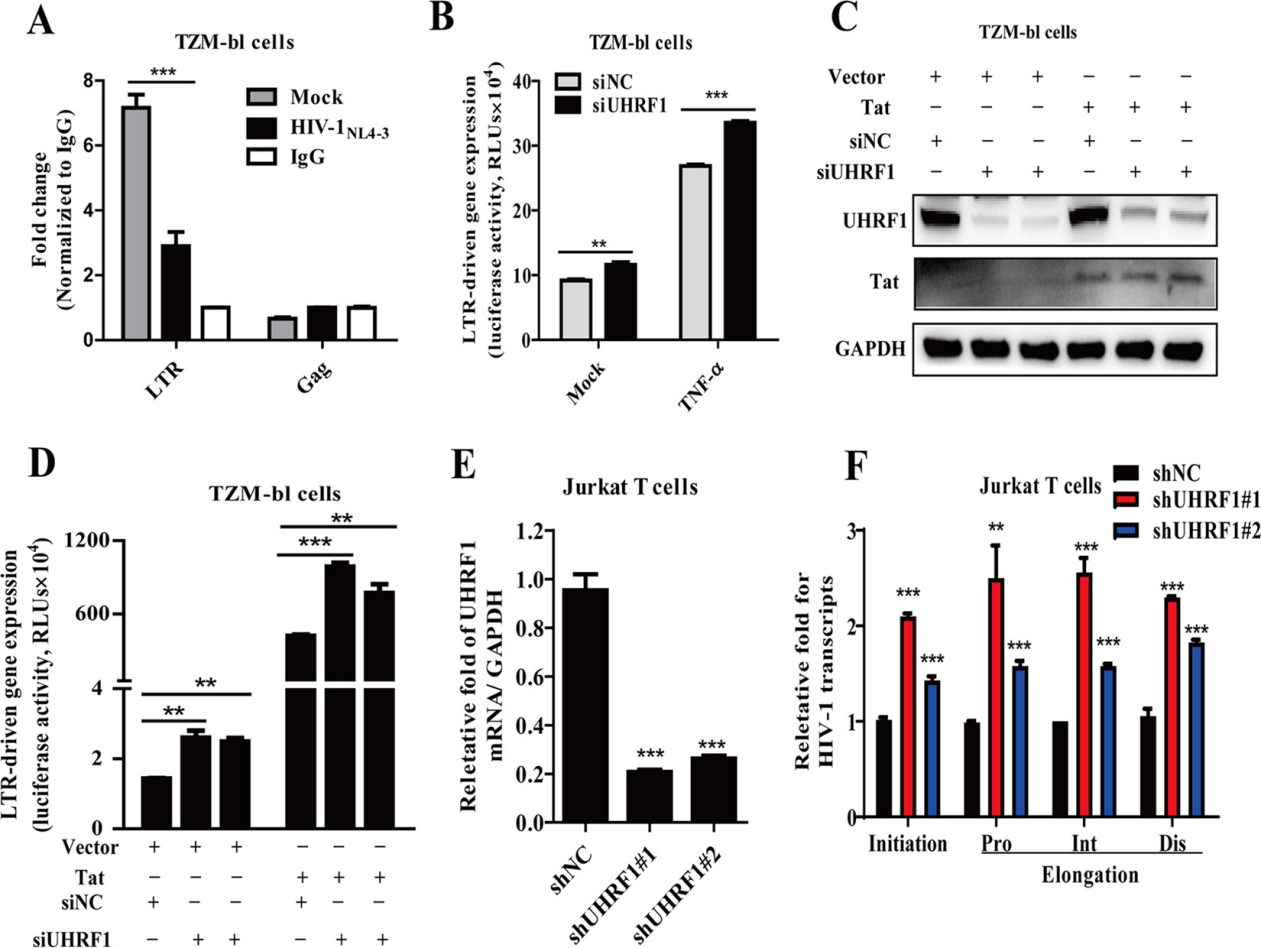
UHRF1 associates with HIV-1 5′-LTR and suppresses viral transcription. (A) TZM-bl cells were infected with HIV-1_NL4-3_ and ChIP-qPCR analysis of the enrichment of UHRF1 at the 5′-LTR promoter, with the integrated coding sequences of the HIV-1 Gag gene as a negative control. All the amplicons first display percent enrichment compared to the input, followed by normalizing to the isotype IgG. (B) TZM-bl cells were stimulated with TNF-α, and 5′-LTR-driven gene expression was monitored by measuring luciferase activity. (C and D) TZM-bl cells stably expressing HIV-1 Tat were transfected with UHRF1 siRNAs. The expression of UHRF1 and Tat was detected by immunoblotting (C), and HIV-1 5′-LTR-driven gene expression was detected by measuring luciferase activity (D). (E) The knockdown efficiency of UHRF1 in Jurkat T cells was detected by RT-qPCR. (F) Jurkat T cells were infected with HIV-1_IIIB_, and transcription initiation (Ini) and proximal (Pro), intermediate (Int), and distal (Dis) elongations were quantified by RT-qPCR with specific primers. Data are representative of the means ± SD from three independent experiments. Statistical significance was determined by unpaired Student's *t* test and one-way ANOVA followed by Dunnett’s multiple-comparison *post hoc* test: **, *P < *0.01; ***, *P < *0.001.

The HIV-1 5′-LTR promoter plays an essential role in driving viral transcription and productive infection (21, 23). Based on the role of UHRF1 in HIV-1 replication, we speculated that UHRF1 is recruited to the HIV-1 5′-LTR promoter. To verify this, we first tested the binding of UHRF1 to the HIV-1 5′-LTR promoter in TZM-bl cells infected with HIV-1_NL4-3_ by chromatin immunoprecipitation (ChIP) assay. Here, the integrated coding sequences of the HIV-1 Gag gene were a reliable negative control. All the amplicons first display percent enrichment compared to the input, followed by normalizing to the isotype IgG. As shown in Fig. 2A, UHRF1 was robustly recruited to HIV-1 5′-LTR, and HIV-1_NL4-3_ infection significantly abolished the recruitment of UHRF1 to HIV-1 LTR. The integrated Gag region did not show the association with UHRF1 (Fig. 2A). Taken together, these data demonstrate that UHRF1 could be recruited to the HIV-1 5′-LTR promoter and antagonize viral transcription.

To assess the importance of the binding of UHRF1 to the HIV-1 5′-LTR, we further investigated the effect of UHRF1 on HIV-1 5′-LTR-driven transcription. Here, we used tumor necrosis factor α (TNF-α), a potent activator of transcription directed by the HIV-1 LTR, to induce HIV-1 LTR-driven gene expression. As shown in Fig. 2B, depletion of UHRF1 modestly increased both basal and stimulated LTR-driven gene expression. The HIV-1 Tat protein binds the TAR region for driving transcription elongation, so we wondered whether the inhibitory effect of UHRF1 on LTR-driven transcription was related to the Tat protein. TZM-bl cells with stable expression of HIV-1 Tat protein were transiently transfected with UHRF1 siRNAs, and the expression levels of Tat and UHRF1 were confirmed by immunoblotting (IB) (Fig. 2C). The results showed that LTR-driven gene expression was significantly increased by Tat overexpression (Fig. 2D). However, UHRF1 depletion increased both basal (by 1.7- to 1.8-fold) and Tat-mediated LTR-driven transcription (by 1.8- to 2.4-fold) compared to that in the corresponding siNC groups (Fig. 2D).

We further investigated the effect of UHRF1 on HIV-1 initiation and elongation in LTR-driven transcription by monitoring reverse transcription-PCR (RT-PCR) products with specific primers targeting initiation, proximal (Pro), intermediate (Int), and distal (Dis) sites of HIV-1 transcripts (24). Our data showed UHRF1 knockdown (Fig. 2E) increased both initiation and elongation in HIV-1 5′-LTR-driven transcription (Fig. 2F). Taken together, these data demonstrate that UHRF1 binds to the HIV-1 5′-LTR for suppression of HIV-1 transcription.

### UHRF1 promotes HIV-1 latency.

**FIG 3:**
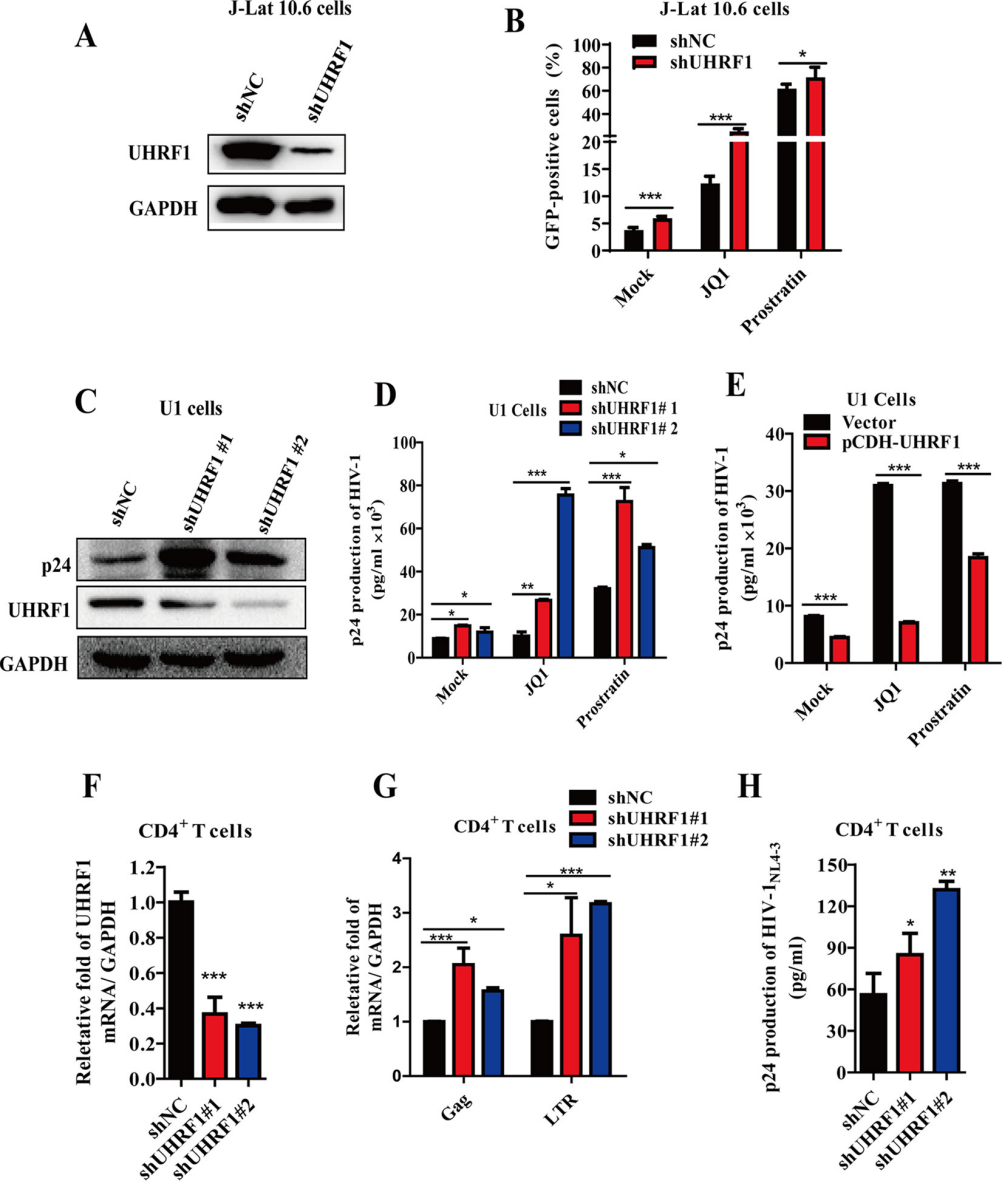
UHRF1 depletion promoted HIV-1 reactivation. (A) J-Lat 10.6 cells were stably transduced with UHRF1 shRNA, and UHRF1 expression was detected by immunoblotting. (B) Cells were treated with JQ1 (1 μM) or prostratin (1 μM) for 48 h, and the percentage of GFP^+^ cells was monitored by flow cytometry. The percentage of GFP^+^ cells was summarized and shown as means ± SD from three independent experiments. (C) UHRF1 was stably depleted in U1 cells with UHRF1-specific shRNA, and the expression of UHRF1 and HIV-1 p24 was detected by immunoblotting. (D) The cells were further stimulated with JQ1 (1 μM) or prostratin (1 μM), and the supernatants were harvested to detect HIV-1 production by Gag p24 capture ELISA. (E) The cells with stably expressed UHRF1 were stimulated with JQ1 (1 μM) or prostratin (1 μM), and the supernatants were harvested to detect HIV-1 production by Gag p24 capture ELISA. (F to H) Resting CD4^+^ T cells were stably depleted of UHRF1 with UHRF1-specific shRNA. The mRNA level of UHRF1 (F) or HIV-1 LTR and Gag (G) was analyzed by RT-qPCR. The cell culture supernatants were harvested to detect HIV-1 production by Gag p24 capture ELISA (H). Data are presented as the means ± SD from three independent experiments. Statistical significance was determined by unpaired Student's *t* test and one-way ANOVA followed by Dunnett’s multiple-comparison *post hoc* test: *, *P < *0.05; **, *P < *0.01; ***, *P < *0.001.

HIV-1 latency is characterized by silencing LTR-driven transcription of integrated HIV-1 proviral DNA. The inhibitory effect of UHRF1 on HIV-1 transcription led us to speculate on the role of UHRF1 in the maintenance of HIV-1 latency. First, the well-established latently infected J-Lat 10.6 cell line, which contains a full-length integrated HIV-1 genome with HIV-1 *Δenv* from the NL4-3 backbone and a green fluorescent protein (GFP) reporter gene in place of the *Nef* gene (25), was infected with shUHRF1 lentiviruses to knock down endogenous UHRF1 (Fig. 3A). The results showed that depletion of UHRF1 promoted HIV-1 reactivation, indicated by an increased percentage of GFP-positive cells on J-Lat 10.6 cells, with a 1.2- to 2-fold increase of reactivation under both unstimulated conditions and stimulation by JQ1 or prostratin (Fig. 3B). Not only the basal level of HIV-1 Gag mRNA but also that upon stimulation with JQ1 or prostratin was potently increased after the depletion of UHRF1 (Fig. S2A). These results were also confirmed in another HIV-1 latency-infected cell line, J-Lat A2 cells (Fig. S2C and D). Furthermore, similar results were observed in a chronically latent HIV-1 U1 cell line, which contains two copies of the latent HIV-1 provirus (26). With UHRF1 depletion in U1 cells (Fig. 3C), the production of HIV-1 p24 in the culture supernatants was also enhanced under basal and LRA stimulation conditions (Fig. 3D). Consistent with these findings, overexpression of UHRF1 in U1 cells conversely decreased the production of HIV-1 p24 under both basal and LRA stimulation (Fig. 3E).

10.1128/mBio.01625-21.2 FIG S2 UHRF1 promotes HIV-1 latency. (A) J-Lat 10.6 cells with UHRF1 knockdown were treated with LRAs, and the levels of HIV-1 Gag mRNA were detected by RT-qPCR. The results were normalized to the expression of GAPDH. (B) The knockdown efficiency of UHRF1 in J-Lat A2 cells was detected by immunoblotting assay. (C)The J-Lat A2 cells were treated with JQ1 (1 μM) or prostratin (1 μM) for 24 h, and the number of GFP^+^ cells was measured by flow cytometry. Total RNA was harvested, and the mRNA levels of HIV-1 Gag were detected by RT-qPCR. (D) The results were normalized to the expression of GAPDH. Data are presented as the means ± SD. **, *P < *0.01; ***, *P < *0.01 (unpaired Student’s *t* test). Download FIG S2, TIF file, 1.3 MB. Copyright © 2021 Liang et al. 2021 Liang et al. https://creativecommons.org/licenses/by/4.0/ This content is distributed under the terms of the Creative Commons Attribution 4.0 International license.

Furthermore, we employed a primary CD4^+^ T cell model of HIV-1 latency as previously described by Lewin’s group (27) to confirm the role of UHRF1 in the maintenance of HIV-1 latency. Resting CD4^+^ T cells were isolated with a positive CD4^+^ T cell selection kit, and the efficiency of shRNA-mediated UHRF1 knockdown was measured by real-time quantitative PCR (RT-qPCR) (Fig. 3F). Importantly, UHRF1 depletion elevated the level of HIV-1 Gag and LTR mRNA and p24 in the culture supernatants in primary CD4^+^ T latent cells (Fig. 3G and H). These results suggest that UHRF1 is a positive regulator for the maintenance of HIV-1 latency, and its depletion benefits HIV-1 latency reactivation under both basal and LRA-stimulated latent HIV-1-infected cells.

### UHRF1 competes with p-TEFb for the binding to Tat.

**FIG 4:**
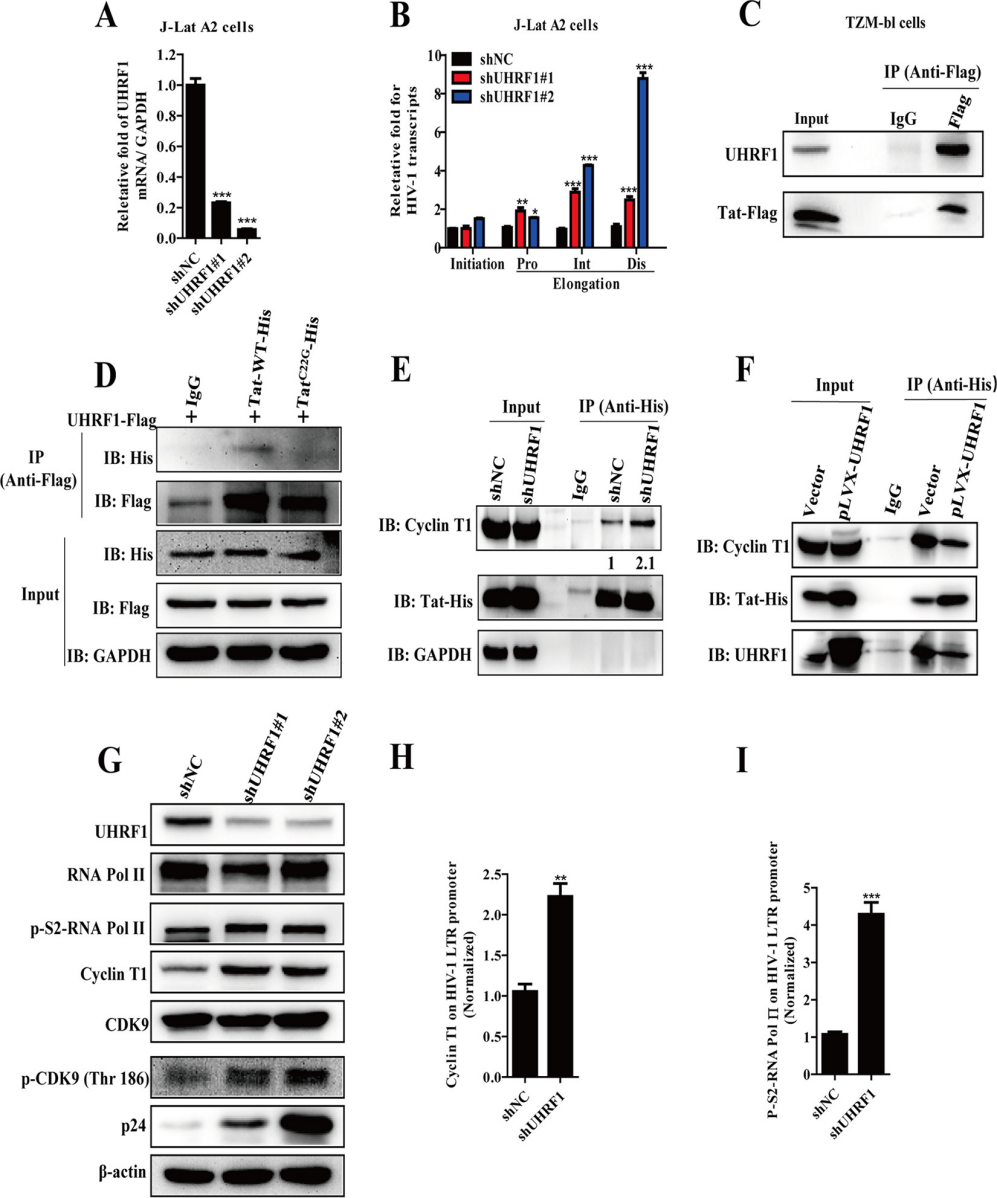
UHRF1 competes with p-TEFb for binding to HIV-1 Tat. (A) J-Lat A2 cells were stably transduced with UHRF1 shRNA, and the cells were harvested for total cellular RNA isolation. UHRF1 expression was detected by RT-qPCR. (B) The transcription initiation (Ini) and proximal (Pro), intermediate (Int), and distal (Dis) elongations of J-Lat A2 cells were quantified with RT-qPCR with specific primers. (C) TZM-bl cells were transfected with HIV-1 Tat-Flag expression plasmid, and the association between the endogenous UHRF1 and HIV-1 Tat was analyzed by coimmunoprecipitation assay. (D) Coimmunoprecipitation assay analysis of the interaction of UHRF1-Flag with Tat-His or the Tat mutation of Cys 22 to Gly-His (Tat^C22G^-His). (E) Coimmunoprecipitation assay was used to examine the association of cyclin T1 and HIV-1 Tat upon depletion of endogenous UHRF1. Quantification of relative cyclin T1 levels is shown in the bottom panel, normalized to IP-Tat-His. (F) The association of cyclin T1 and HIV-1 Tat upon transient transfection with a plasmid for UHRF1 expression in 293T cells was analyzed by coimmunoprecipitation assay. (G) The expression levels of CDK9, p-CDK9 (Thr 186), cyclin T1, RNA Pol II, P-S2-RNA Pol II, and p24 were analyzed by immunoblotting in NC and UHRF1-depleted U1 cells. (H and I) The association of cyclin T1 (H) or phosphorylated RNA Pol II (I) with the HIV-1 5′-LTR was measured by ChIP-PCR assay using LTR-specific primers. Data are presented as the means ± SD from three independent experiments. Statistical significance was determined by unpaired Student's *t* test or one-way ANOVA followed by Dunnett’s multiple-comparison *post hoc* test: *, *P < *0.05; **, *P < *0.01; ***, *P < *0.001.

To further decipher the underlying mechanism(s) by which UHRF1 promotes HIV-1 latency, we explored the effects of UHRF1 depletion on the levels of initiated and elongated HIV-1 transcription in latent HIV-1 J-Lat A2 cells. Surprisingly, depletion of UHRF1 (Fig. 4A) in J-Lat A2 cells had no effect on the initial HIV-1 transcripts but significantly increased HIV-1 elongation, including the proximal, intermediate, and distal HIV-1 transcripts (Fig. 4B). Notably, J-Lat A2 cells highly expressing the Tat protein are a well-established T lymphocyte cell model of HIV latency derived from human Jurkat T cells. Taking into account that Tat, which interacts with and activates cellular p-TEFb (28), plays a more important role in regulating HIV-1 transcriptional elongation than in its initiation (29), we speculated that Tat is involved in UHRF1-mediated HIV-1 transcriptional elongation and HIV-1 latency.

To explore the role of HIV-1 Tat in UHRF1-mediated HIV-1 latency, the physiological interaction between viral Tat protein and UHRF1 was investigated by coimmunoprecipitation assays. The data showed that both endogenous (Fig. 4C) and exogenous (Fig. S3A) UHRF1 potently associated with Tat. In line with the results described above, the immunofluorescence assay further confirmed that UHRF1 was colocalized with HIV-1 Tat in the nucleus (Fig. S3C). Considering the importance of Tat ubiquitination and Tat-cyclin T1 interaction for Tat transactivation, we constructed a panel of Tat mutants to screen the key residues that are possibly involved in the binding to UHRF1. Coimmunoprecipitation assays demonstrated that mutations of lysine (Lys) residue 19 or 71 to arginine in Tat protein (Tat^K19R^ and Tat^K71R^) did not affect the interaction of Tat with UHRF1; however, the Tat^K29R^ mutant reduced the Tat-UHRF1 interaction (Fig. S3B). Furthermore, we found that mutation of cysteine 22 (Cys 22) to glycine (Gly) in the Tat activation domain (Tat^C22G^) also failed to bind to UHRF1 (Fig. 4D), indicating that Cys 22 and Lys 29 in the cysteine-rich domain of Tat are the accountable attachment sites for the interaction of Tat with UHRF1. Since the Tat mutant in which Cys 22 was replaced by Gly also failed to bind to cyclin T1 (30), we further investigated the effect of UHRF1 on the interaction between the p-TEFb complex (containing CDK9 and cyclin T1) and Tat. The depletion of UHRF1 increased the association of Tat with cyclin T1 in coimmunoprecipitation (co-IP) assays (Fig. 4E), and the overexpression of UHRF1 conversely decreased the interaction (Fig. 4F), indicating that UHRF1 is competing with p-TEFb for the binding to Tat. Furthermore, we found that UHRF1 knockdown in U1 cells increased the expression of cyclin T1 and phosphorylation of CDK9 at Thr186, required for the catalytic activity of CDK9 (Fig. 4G). UHRF1 knockdown also increased the occupancy of cyclin T1 at the HIV-1 5′-LTR, as determined by ChIP-PCR assay (Fig. 4H). Since the p-TEFb complex phosphorylates Ser2 at the CTD of RNA Pol II for subsequent transcriptional elongation, we further examined the effects of UHRF1 on RNA Pol II. UHRF1 knockdown increased the phosphorylation of RNA Pol II (Fig. 4G) and resulted in a significant 4.3-fold increase in the occupancy of RNA Pol II at the HIV-1 5′-LTR (Fig. 4I). Taken together, our results demonstrate that UHRF1 interacts with HIV-1 Tat by competing with p-TEFb, leading to the blockade of transcription elongation.

10.1128/mBio.01625-21.3 FIG S3 Physiological interaction between viral Tat protein and UHRF1. (A) Immunoprecipitation analysis of HEK293T cells transfected with Tat-Flag along with PCDH-UHRF1, followed by IP with anti-Flag, probed with anti-UHRF1 antibody. (B) Immunoprecipitation analysis of HEK293T cells transfected with UHRF1-Flag along with Tat-His or the Tat mutants (Tat^K19R^, Tat^K29R^, and Tat^K71R^), followed by IP with anti-Flag, probed with anti-His antibody. (C) Confocal microscopy of TZM-bl cells transfected with Tat-His and UHRF1 WT-Flag expression plasmids. Scale bars represent 10 μm. (D) J-Lat A2 cells were treated with NSC232003 in different concentrations and prostratin (1 μM) for 48 h, and the percentage of GFP^+^ cells was monitored by flow cytometry. The percentage of GFP^+^ cells was summarized and shown as mean ± SD from three independent experiments. (E) U1 cells were stimulated with various concentration of NSC232003 for 48 h, and the supernatants were harvested to detect HIV-1 production by Gag p24 capture ELISA. Download FIG S3, TIF file, 3.0 MB. Copyright © 2021 Liang et al. 2021 Liang et al. https://creativecommons.org/licenses/by/4.0/ This content is distributed under the terms of the Creative Commons Attribution 4.0 International license.

### The RING domain of UHRF1 is essential for HIV-1 suppression.

**FIG 5:**
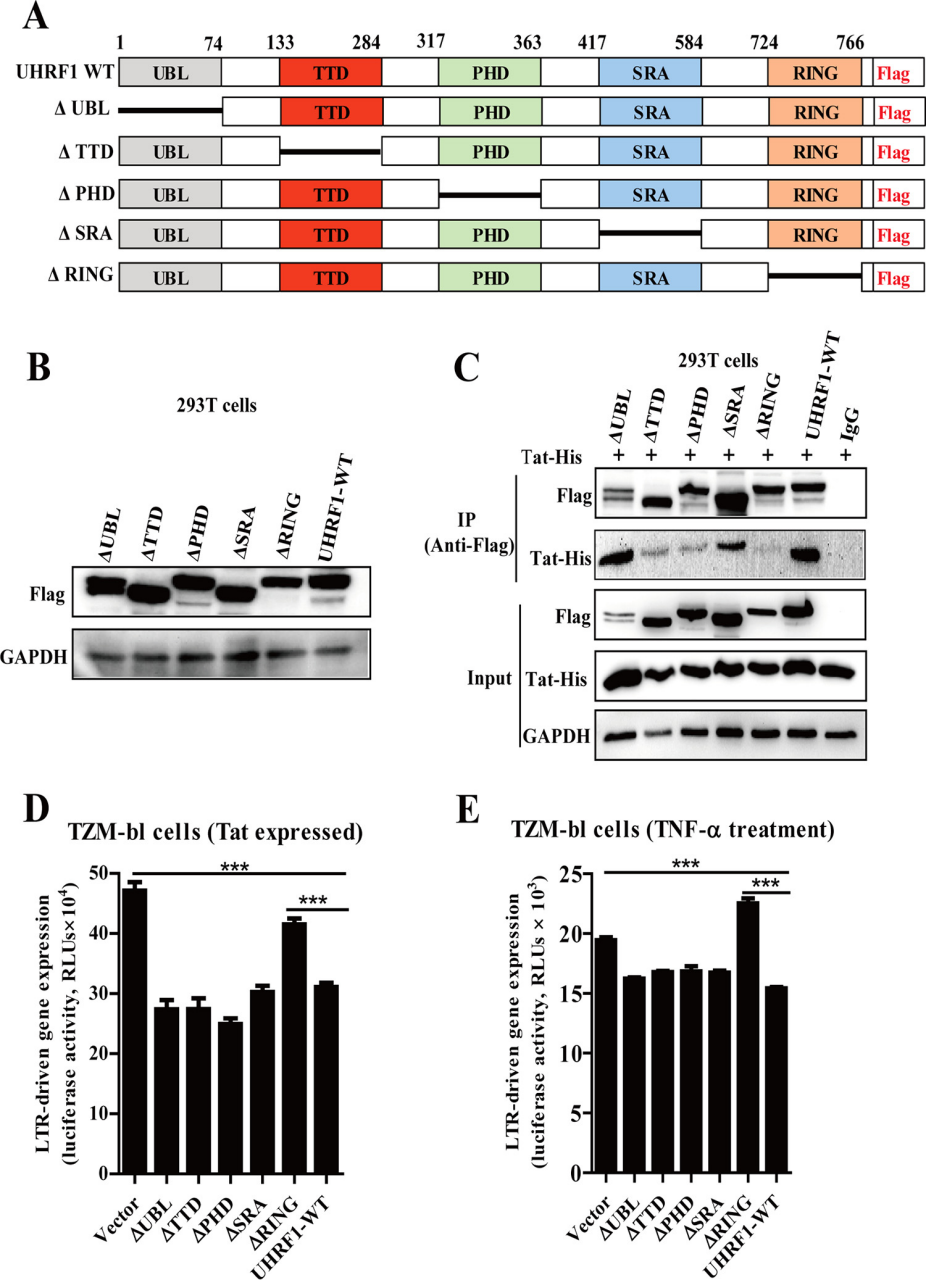
RING finger domain of UHRF1 is crucial for HIV-1 suppression. (A) Schematic diagram showing the wild-type UHRF1 protein and UHRF1 mutants. A Flag-tagged element was added to the C terminus of the constructs. (B) HEK293T cells were transiently transfected with the indicated plasmids, and the cellular lysates were analyzed by immunoblotting. (C) HEK293T cells were transfected with the indicated plasmids, the cell lysates were subjected to immunoprecipitation by using an anti-Flag antibody or an IgG control, and immunoblotting was performed. (D) TZM-bl cells stably expressing HIV-1 Tat were transfected with the indicated plasmids for 48 h, and HIV-1 5′-LTR-driven gene expression was detected by measuring luciferase activity. (E) TZM-bl cells stimulated with TNF-α were transfected with the indicated plasmids, and 5′-LTR-driven gene expression was monitored by measuring luciferase activity. Data are presented as means ± SD from three independent experiments. Statistical significance was determined by one-way ANOVA followed by Dunnett’s multiple-comparison *post hoc* test: ***, *P < *0.001.

Considering the importance of the association of UHRF1 with Tat, we were interested in identifying the major structural domain(s) of UHRF1 critical for mediating the interaction. UHRF1 is a multifunctional protein containing five domains (ubiquitin-like [UBL] domain, tandem Tudor domain [TTD], plant homeodomain [PHD], SET- and RING-associated [SRA] domain, and RING domain). Based on the structural analysis, five Flag-tagged deletion mutants of UHRF1 were constructed (Fig. 5A), and their expression efficiencies were comparable, as determined by immunoblotting (Fig. 5B). The co-IP results showed that the ΔTTD (deletion mutant lacking amino acids 133 to 284), ΔPHD (deletion mutant lacking amino acids 317 to 363), and ΔRING finger domain of UHRF1 (deletion mutant lacking the C-terminal amino acids 724 to 766) obviously disrupted the association of UHRF1 with Tat (Fig. 5C). Other deletion mutants, including ΔUBL and ΔSRA of UHRF1, were dispensable for the UHRF1-Tat interaction (Fig. 5C). We speculate that several key amino acid residues from TTD, PHD, and RING domains constitute one binding motif to interact with Tat, which needs to be further proven via crystal structure analysis of the UHRF1-Tat complex in the future.

Furthermore, the TZM-bl indicator cells stably expressing HIV-1 Tat protein or stimulated with TNF-α were transiently transfected with the UHRF1 wild type (WT) and five UHRF1 mutants. Tat- or TNF-α-mediated HIV-1 5′-LTR-driven gene expression was significantly decreased in response to the overexpression of WT UHRF1 or the ΔUBL, ΔTTD, ΔPHD, and ΔSRA truncated mutants (Fig. 5D and E). Intriguingly, overexpression of the ΔRING finger domain mutant abolished the inhibitory effect of UHRF1 on HIV-1 5′-LTR-driven gene expression (Fig. 5D and E), suggesting the critical role of the RING finger domain in UHRF1-mediated HIV-1 suppression. It has been reported that the RING finger domain of UHRF1 recruits DNA methyltransferase DNMT1 to DNA replication sites for the maintenance of DNA methylation. To explore whether UHRF1-mediated HIV-1 suppression relies on the recruitment of DNMT1 and DNA methylation, a small-molecule UHRF1 inhibitor, NSC232003, was incubated with latently infected cell lines J-Lat A2 and U1. As an effective DNA methylation inhibitor, NSC232003 potently disrupts the DNMT1/UHRF1 interaction with a 50% infective concentration (IC_50_) of 15 μM (31). Our results showed that NSC232003 treatment did not affect HIV-1 latency in J-Lat A2 (Fig. S3D) and U1 cells (Fig. S3E), indicating that the effect of UHRF1 on HIV-1 latency is independent of the regulation of DNA methylation. Overall, these data suggest that TTD, PHD, and RING finger domains are important for the UHRF1-Tat interaction, partially impaired via the deletion of three domains separately; however, the interaction of the RING finger motif with HIV-1 Tat is pivotal for mediating HIV-1 suppression. We speculate that UHRF1-mediated HIV-1 suppression requires the RING domain for further modification of Tat after the UHRF1-Tat interaction.

### UHRF1 promotes ubiquitination and proteasomal degradation of HIV-1 Tat.

**FIG 6:**
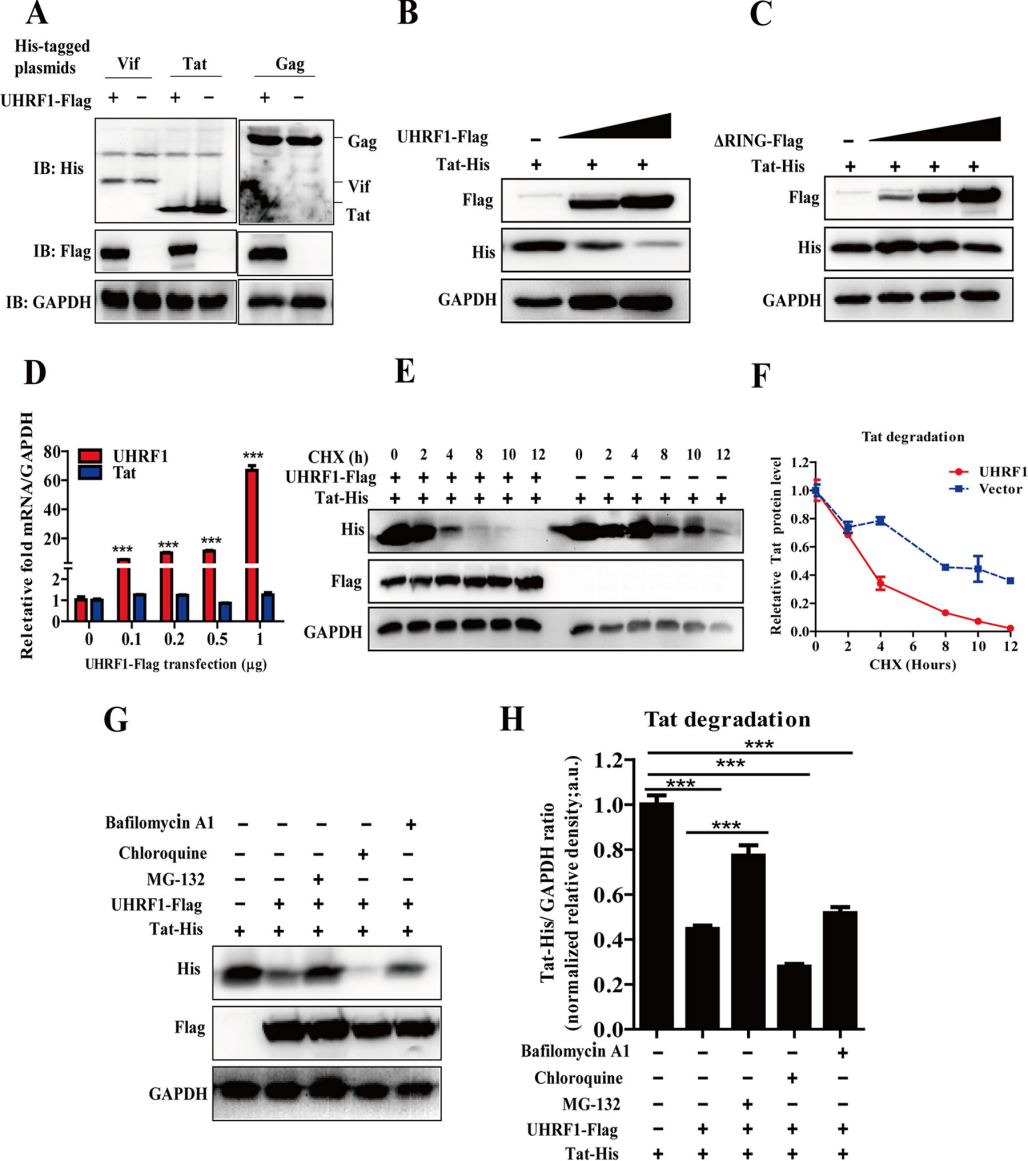
UHRF1-mediated HIV-1 Tat degradation in proteasome. (A) Immunoblot analysis of HEK293T cells cotransfected with indicated His-tagged plasmids expressing HIV-1 viral protein (Vif, Tat, and Gag) and a Flag-tagged plasmid expressing UHRF1. (B and C) Immunoblot analysis of HEK293T cells cotransfected with a His-tagged plasmid expressing Tat and a Flag-tagged plasmid expressing UHRF1 (B) or the ΔRING mutant of UHRF1 (C) with different concentrations. (D) RT-PCR analysis of the mRNA expression of UHRF1 and Tat in HEK293T cells transfected with a His-tagged plasmid expressing Tat (2 μg) and a Flag-tagged plasmid expressing UHRF1 with different concentrations. (E and F) Immunoblot analysis of HEK293T cells cotransfected with a His-tagged plasmid expressing Tat (2 μg) and a Flag-tagged plasmid expressing UHRF1 (2 μg) in the treatment of cycloheximide (CHX, 20 μM) at different time points (E). The expression of Tat protein was quantitated by measuring band intensities using ImageJ software (F). The values were normalized to GAPDH. (G and H) Immunoblot analysis of HEK293T cells cotransfected with a His-tagged plasmid expressing Tat (2 μg) and a Flag-tagged plasmid expressing UHRF1 (2 μg) in the treatment of MG-132 (20 μM), chloroquine (100 μM), and bafilomycin A1 (20 μM) for 4 h (G). The expression of Tat protein was quantitated by measuring band intensities using ImageJ software (H). The values were normalized to GAPDH. Data are presented as means ± SD from three independent experiments. Statistical significance was determined by unpaired Student's *t* test or one-way ANOVA followed by Dunnett’s multiple-comparison *post hoc* test: ***, *P < *0.001.

The RING domain plays a key role in the E3 ubiquitin ligase activity of UHRF1. Thus, it is conceivable that the RING finger domain promotes Tat ubiquitination and degradation after interacting with HIV-1 Tat. We next investigated whether UHRF1 could modulate Tat protein stability. UHRF1 overexpression resulted in reduced levels of Tat but not other viral proteins (Fig. 6A). Intriguingly, UHRF1 triggered the degradation of Tat in HEK293T cells in a dose-dependent manner (Fig. 6B), with no variation in Tat mRNA levels in response to increasing UHRF1 expression (Fig. 6D), clearly suggesting that UHRF1 promotes Tat degradation at the posttranslational level. In addition, the ΔRING finger domain mutant lost the ability to promote UHRF1 degradation (Fig. 6C). Finally, the treatment of cycloheximide (CHX), a protein synthesis inhibitor, dramatically decreased the half-life of Tat with UHRF1 overexpression, indicating that UHRF1 modulates the stability of Tat (Fig. 6E and F). These results confirmed that UHRF1 promoted the degradation of Tat protein without affecting its mRNA transcriptional level.

To elucidate the mechanism involved in the regulation of Tat degradation via UHRF1, HEK293T cells were cotransfected with Tat and UHRF1, followed by treatment with different inhibitors, including proteasome inhibitor MG-132 or lysosome/autophagy inhibitor chloroquine and bafilomycin A1. The data showed UHRF1-mediated Tat degradation was blocked by MG-132 but not chloroquine and bafilomycin A1 (Fig. 6G and H), although these drugs inhibited the replication of vesicular stomatitis virus (7).

**FIG 7:**
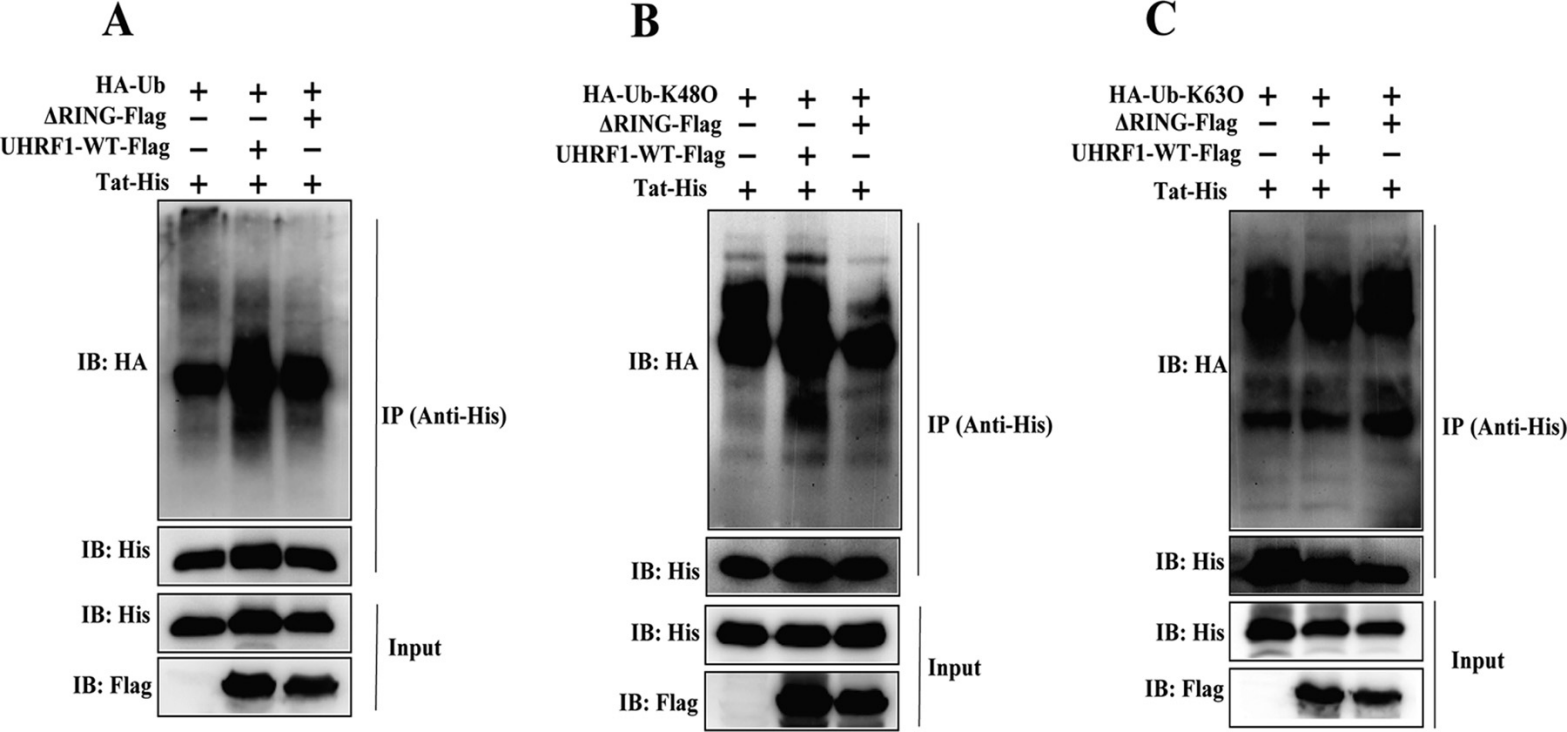
UHRF1 promotes K48-linked ubiquitination of HIV-1 Tat. HEK293T cells were transiently cotransfected with HA-tagged ubiquitin (HA-Ub) (A), HA-tagged K48-linked ubiquitin (HA-Ub-K48O) (B), and HA-tagged K63-linked ubiquitin (HA-Ub-K63O) (C), a His-tagged plasmid expressing Tat and a Flag-tagged plasmid expressing UHRF1 or the ΔRING mutant of UHRF1. The ubiquitination level of Tat was detected by immunoprecipitation with anti-His antibody and probed with anti-HA antibody by immunoblotting.

Ubiquitination modification is a key step in protein degradation via the ubiquitin-proteasome degradation pathway. We thereby examined the effects of UHRF1 on Tat ubiquitination. Exogenous UHRF1 expression substantially enhanced the ubiquitination level of Tat, which was attenuated with the deletion of the RING finger domain, suggesting that RING finger domain serving as the E3 ubiquitin ligase activity of UHRF1 is critical for Tat ubiquitination (Fig. 7A). In order to further investigate the types of ubiquitin chain linkages on Tat protein catalyzed by UHRF1, hemagglutinin (HA)-Ub-K48O, and HA-Ub-K63O plasmids expressing HA-tagged Lys-48- and Lys-63-only lysine residue ubiquitin (all lysine residues except Lys-48 or Lys-63 are mutated) were used in the ubiquitination assays. UHRF1 WT, but not the ΔRING finger domain mutant, markedly enhanced K48-linked ubiquitination of Tat (Fig. 7B), with no effects on K63-linked ubiquitination of Tat (Fig. 7C). Collectively, these data support a model in which UHRF1 selectively promotes HIV-1 Tat protein ubiquitination, leading to the proteasome degradation of Tat via its E3 ubiquitin ligase activity.

## DISCUSSION

Distinct classes of LRAs have been shown to target different subpopulations of proviruses (22, 25, 32, 33). Thus far, no clinically tested LRAs are able to intensely induce viral expression or diminish the latent reservoir in patients (2, 34, 35). Accumulating evidence demonstrates the critical role of host factors in regulating HIV-1 latency via silencing HIV-1 viral transcription, which are essential for the development of rational therapeutics for the eradication of latent virus (4, 36–38). In this study, we found that UHRF1 plays a key role in the regulation of HIV-1 transcription and replication. Our studies revealed that UHRF1 blocked HIV-1 replication through suppressing LTR-driven HIV-1 transcription. In addition, we further confirmed that UHRF1 promoted HIV-1 latency and prevented HIV-1 reactivation in multiple latently infected cell lines as well as a primary CD4^+^ T cell model. As expected, UHRF1 depletion not only reversed HIV-1 latency but also synergized with multiple LRAs to promote HIV-1 reactivation.

It has been well documented that the intracellular Tat circuit plays a central role in controlling latency and transcription in HIV-infected cells by recruiting p-TEFb and promoting RNA Pol II pause-release and elongation during HIV-1 transcription (7, 39). Moreover, recent studies showed that several host factors associated with the HIV-1 transactivator Tat for the suppression of viral transcription and establishment of HIV-1 latency (13, 14). Interestingly, we found that UHRF1 preferentially blocked HIV-1 transcription elongation in latent cell lines. Mechanistically, our work demonstrated that Tat was associated with either endogenous or exogenous UHRF1. Tat interacts with cyclin T1, one component of the p-TEFb complex, to facilitate HIV-1 transcriptional elongation via its Cys 22 residue, located in the Tat activation domain. Our data also showed that mutation of Cys 22 to Gly (Tat^C22G^) abrogated the interaction with UHRF1, indicating that UHRF1 competes with cyclin T1 for binding to the Cys 22 site of Tat. As expected, the loss of UHRF1 enhanced Tat-cyclin T1 interaction, leading to the increase of cyclin T1 recruitment to the HIV-1 LTR and RNA Pol II CTD phosphorylation and viral transcription. Meanwhile, a Tat^K29R^ mutant reduced the Tat-UHRF1 interaction with Tat and UHRF1, suggesting a role of the Lys 29 residue in the ubiquitination-proteasome degradation of Tat by UHRF1.

Truncated mutants of UHRF1 were constructed for screening the domains of UHRF1 involved in the interaction with Tat. UHRF1 consists of five recognizable functional domains: the UBL domain at its N terminus, followed by the TTD domain that binds to H3K9me2/3, the PHD domain that binds to the histone H3 tail, the SRA domain that recognizes hemimethylated CpG-containing DNA, and the RING finger domain at its C terminus that confers UHRF1 with intrinsic E3 ubiquitin ligase activity (40, 41). The RING finger domain is a specialized type of Zn finger 40 to 60 residues in length that binds two zinc atoms. Zn fingers are probably involved in mediating protein-protein interactions, signal transduction, and viral replication (42). Accordingly, the truncated mutants of UHRF1 were constructed for screening the domains of UHRF1 involved in the interaction with Tat. The partial impairment of UHRF1-Tat interaction was observed by deleting the TTD, PHD, or C-terminal RING finger domain, respectively. Similar binding patterns have been reported, showing that UHRF1 recognizes histone H3 Lys 9 (H3K9) and Lys 23 (H3K23) via its TTD-PHD and RING domains, respectively (18, 43, 44). Surprisingly, the loss of RING domain, rather than TTD or PHD domain, abolished the inhibitory effect of UHRF1 on HIV-1 5′-LTR-driven gene expression, suggesting that the RING domain is required for further modification of Tat. Recent studies suggest that viruses interact with the host RING domain E3 ligase to modulate viral replication through the ubiquitination of viral proteins (45–47), and the RING domain responsible for E3 ubiquitin ligase activity of UHRF1 promotes the ubiquitination of H3K23 (44). Intriguingly, we showed that the RING finger domain interacted with HIV-1 Tat and further promoted K48-linked ubiquitination and proteasomal degradation of Tat. This finding provides new insights into the regulation of Tat degradation; thus, controlling the Tat expression by UHRF1 is a potential therapeutic strategy for the intervention of HIV-1 or regulation of HIV-1 latency reactivation. It is well known that UHRF1 plays a critical role in the maintenance of DNA methylation, which relies on the recognition of H3K9me3 and hemimethylated CpG DNA and recruitment of DNA methyltransferase Dnmt1 via its TTD-PHD, SRA, and RING domains. Of note, the loss of the TTD, PHD, or SRA domain had no effect on UHRF1-mediated suppression of viral gene expression, indicating that the effect of UHRF1 on HIV-1 transcription and latency is independent of UHRF1-mediated DNA methylation. Moreover, specific inhibitor NSC232003, which modulates DNA methylation by disrupting DNMT1/UHRF1 interaction (31), did not show the reactivation effect on the HIV-1 latency.

**FIG 8:**
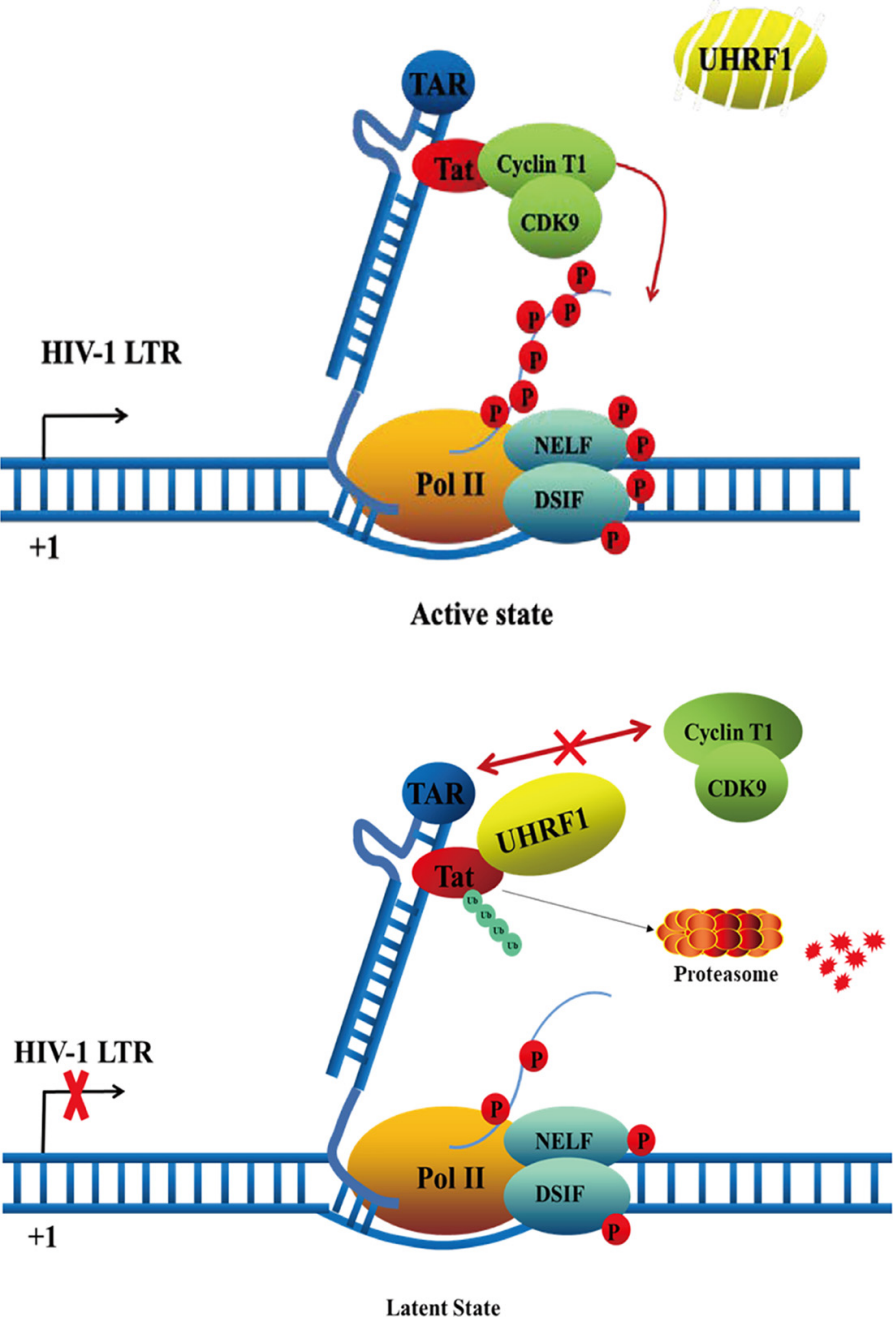
Schematic of the molecular mechanisms underlying the function of UHRF1 in HIV-1 proviral expression. The HIV-1 Tat protein is a critical viral factor that recruits host positive transcription elongation complex b (p-TEFb), composed of cyclin T1 and CDK9, to activate RNA polymerase II (Pol II) for HIV-1 transcriptional elongation. The binding of UHRF1 with Tat competes with p-TEFb and promotes Tat degradation in the ubiquitin-proteasome pathway and, thus, prevents the recruitment of Tat and p-TEFb as well as the activation of RNA Pol II.

In conclusion, we demonstrated a novel role of UHRF1 in the regulation of HIV-1 transcription and latency. Based on our data, we propose a model of UHRF1-mediated HIV-1 latency. In the active state, the HIV-1 Tat protein hijacks the p-TEFb complex to the transcribed HIV-1 RNA TAR. The p-TEFb catalytic subunit CDK9 then superphosphorylates the Ser2 residue of RNA Pol II, facilitating the processivity of RNA Pol II in the transcription of HIV-1 RNA. In the latent state, UHRF1 interacts with HIV-1 Tat by competing with p-TEFb and disrupts the Tat/p-TEFb complex through mediating K48-linked ubiquitination and proteasomal degradation of Tat, leading to blockade of the phosphorylated RNA Pol II and consequential impediment of transcription elongation (Fig. 8). Our findings provide important insights on host-pathogen interaction for controlling HIV-1 transcription and latency.

## MATERIALS AND METHODS

### Cell culture.

HEK293T and TZM-bl cells were obtained from the American Type Culture Collection and cultured at 37°C in Dulbecco’s modified Eagle medium supplemented with 1% l-glutamine (Gibco, Life Technologies), 10% fetal bovine serum (FBS; ExCell Bio, China), and 1% penicillin-streptomycin (Gibco, Life Technologies). J-Lat cell lines (10.6 and A2), Jurkat T cells, and U1 cells were obtained from the NIH AIDS reagent program and maintained in RPMI 1640 medium (Gibco, Life Technologies) supplemented with 10% FBS and 1% penicillin-streptomycin at 37°C with 5% CO_2_.

### Plasmids, siRNAs, and shRNAs.

The cDNA coding sequences of full-length UHRF1 and HIV-1 Tat were amplified by reverse transcription-PCR, with mRNA from J-Lat A2 lymphocytes as the template. The cDNAs were subcloned into pLVX-IRES-Puro and LV242 vectors. UHRF1 mutants (ΔUBL, ΔTTD, ΔPHD, ΔSRA, and ΔRING) and mutation of Tat (Tat^K19R^, Tat^K29R^, Tat^K71R^, and Tat^C22G^) were constructed into pLVX-IRES-Puro vector using an overlapping PCR. All constructs were confirmed by DNA sequencing. Expression plasmids HA-Ub, HA-Ub-K48O, and HA-Ub-K63O were maintained in our lab. A mixture of siRNAs used for UHRF1 knockdown was synthesized (GenePharma, Suzhou, China). Lipofectamine 2000 (Invitrogen) was used for siRNA and plasmid transfection according to the manufacturer’s protocol. The shRNAs targeting UHRF1 and off-target shRNA were subcloned into the pLKD-CMV-mCherry-2A-Puro-U6-shRNA vector, and the sequences are listed in Table 1. PolyJet-mediated transfection of HEK293T cells was used to generate lentiviruses containing UHRF1 shRNA or a plasmid for UHRF1 or HIV-1 Tat expression.

**TABLE 1:** List of primers for construction of shRNAs and siRNAs

Target gene or name	Sequence of siRNA or shRNA primer
siUHRF1#1	GCCAUACCCUCUUCGACUATT
siUHRF1#2	GCAUCUACAAGGUUGUGAATT
siNC	UUCUUCGAACGUGUCACGUTT
shUHRF1#1	GCCAGAGTGAGTCAGACAA
shUHRF1#2	GCTGGCTCTCAACTGCTTT
shNC	TTCTCCGAACGTGTCACGT

### Stable cell lines.

Lentiviral stocks were produced in HEK293T cells cotransfected with 400 ng of vesicular stomatitis virus glycoprotein-expression vector, 600 ng of lentiviral packaging construct psPAX2 and 1 μg of lentiviral vector expressing target genes (Tat and UHRF1) or shRNAs by using PolyJet (SignaGen) according to the manufacturer’s instructions. Lentivirus supernatants were collected by centrifuging at 1,500 rpm for 15 min to remove debris. For virus transduction, cell lines (HEK293T, Jurkat T, TZM-bl, J-Lat 10.6, J-Lat A2, U1, PBMC, and CD4^+^ T cells) were seeded in 12-well plates and spin infected with lentivirus and cultured for 48 h, followed by puromycin (Sigma-Aldrich) selection. Three days postselection, the supernatant of infected cells were replaced with fresh medium and infected cells continued to culture for 2 to 7 days. The overexpression or knockdown efficiency was confirmed by Western blotting.

### Virus stock and infection assay.

Replication-competent HIV-1_NL4-3_ viruses were generated by PolyJet-mediated transfection of HEK293T cells with the pNL4-3 plasmid as described previously (48). The plasmid was maintained in our laboratory. Harvested supernatants that contained viral particles were filtered and quantified by Gag p24 capture ELISA. Cells were infected with the replication-competent virus HIV-1_NL4-3_ for the indicated duration, and, after washing, the cells were further cultured for 2 days. Viral infection was detected by measuring the luciferase activity from the cell lysates or detecting viral production in the supernatants by Gag p24 capture ELISA. HIV-1 production was also titrated in TZM-bl indicator cells that contain an LTR-driven luciferase reporter. Briefly, equal amounts of cell culture supernatant containing replication-competent HIV-1_NL4-3_ were used to infect TZM-bl cells for 48 h, and HIV-1 infection was detected by measuring the luciferase activity.

### RT-qPCR.

Total mRNA was extracted using an RNA isolation kit (Foregene, China) and reverse transcribed with PrimeScript RT master mix (TaKaRa, Japan). PCR was performed using SYBR select master mix (TaKaRa) on a LightCycler 480 under the following conditions: 95°C for 15 s for initial denaturation, followed by 40 cycles of 95°C for 15 s and 60°C for 1 min for annealing and extension, respectively. The mRNA levels of genes were normalized to the glyceraldehyde-3-phosphate dehydrogenase (GAPDH) gene as the reference control. Relative gene expression levels were determined using the 2^−ΔΔ^*^CT^* method. The sequences of the primers used for PCR amplification are listed in Table 2.

**TABLE 2:** List of primers for RT-qPCR and ChIP

Primer name	Forward (5′–3′)	Reverse (5′–3′)
Prox	TGGGAGCTCTCTGGCTAACT	TGCTAGAGATTTTCCACACTGA
Int	GTAATACCCATGTTTTCAGCATTATC	TCTGGCCTGGTGCAATAGG
Dis	GAGAACTCAAGATTTCTGGGAAG	AAAATATGCATCGCCCACAT
Initiation	GTTAGACCAGATCTGAGCCT	GTGGGTTCCCTAGTTAGCCA
UHRF1	AAATGGCCTCAAGGGGACTC	CACTTGCACGTGACTTCGTG
Gag	GTCCAGAATGCGAACCCAGA	GTTACGTGCTGGCTCATTGC
LTR	GCCTCCTAGCATTTCGTCACAT	GCTGCTTATATGTAGCATCTGAGG
LTR ChIP	TGGATCTACCACACACAAGG	GGTACTAGCTTGAAGCACCATCC
GAPDH	CTCTGCTCCTCCTGTTCGAC	AGTTAAAAGCAGCCCTGGTGA

### Immunoprecipitation and immunoblotting.

HEK293T or TZM-bl cells transfected with UHRF1 siRNA or off-target controls and/or HIV-1 Tat plasmid were seeded in a 6-well plate and incubated for 48 h. After washing 2 times, the cells were lysed for 30 min on ice. After centrifugation at 12,000 × *g* for 15 min, the lysates were incubated with the indicated antibody at 4°C overnight, 30 μl of protein A/G agarose then was added, and the mixture was further incubated at 4°C for 4 h. The immunoprecipitates were eluted by boiling with SDS sample buffer after washing five times and analyzed by immunoblotting.

For immunoblotting, cells were lysed for 1 h at 4°C in ice-cold radioimmunoprecipitation assay buffer. After centrifugation for 10 min at 12,000 × *g*, the supernatant was boiled in SDS sample loading buffer and analyzed by SDS-PAGE. Specific primary antibodies were used, followed by the use of horseradish peroxidase-conjugated goat anti-mouse IgG or goat anti-rabbit IgG (Sigma) as the secondary antibody. A total of 5% of the lysates was used as the input.

### Ubiquitination assay.

HEK293T cells were transiently cotransfected with HA-Ub and Tat-His along with UHRF1-Flag or ΔRING-Flag for 48 h and then treated with MG-132 (20 μM) for 4 h. Cells were harvested and lysed in M2 buffer (20 mM Tris, pH 7.6, 250 mM NaCl, 3 mM EDTA, 3 mM EGTA, 0.5% Nonidet P-40, 1 mM dithiothreitol) supplemented with 10 mM *N*-ethylmaleimide, protease inhibitor cocktail, and phenylmethylsulfonyl fluoride. Cell extracts were subsequently added with SDS to a final concentration of 1% and boiled for 5 min at 100°C, and diluted to 0.1% SDS with M2 buffer before immunoprecipitation.

### Confocal microscopy.

TZM-bl cells were seeded into 35-mm dishes with 14-mm glass-bottom wells and transfected with the indicated plasmids. At 48 h posttransfection, cells were washed 3 times with phosphate-buffered saline (PBS) and fixed with 4% formaldehyde (Sigma-Aldrich) for 15 min. Subsequently, the cells were washed 3 times with PBS, permeabilized for 5 min with 0.1% Triton X-100, and blocked with 3% bovine serum albumin (BSA) for 1 h. Specific antibodies for Flag tag and His tag were incubated with cells overnight at 4°C, followed by incubation with secondary antibodies for 1 h. Finally, cells were stained with 4′, 6′-diamodino-2-phenylindole (DAPI) (Invitrogen). The specimens were viewed using a FluoView FV3000 confocal microscope (Olympus, Tokyo, Japan).

### ChIP.

ChIP experiments were performed according to the manufacturer’s instructions (Millipore). Briefly, TZM-bl or U1 cells with UHRF1 knockdown were used. In some tests, cells were further infected with the HIV-1_NL4-3_ virus. Cells were cross-linked with 1% formaldehyde for 10 min at room temperature and quenched with 125 mM glycine for 5 min. After washing three times, the cells were lysed and chromatin was sheared by use of a sonicator for a total of 12 rounds (40 output and 10 s/round) to obtain DNA fragments of 200 to 400 bp. One percent of the total sheared chromatin DNA was used as the input sample. Other sheared chromatin was incubated overnight at 4°C with an antibody against UHRF1 (Santa Cruz), cyclin T1 (Santa Cruz), pSer-2 RNA Pol II (CST), or mouse IgG (Proteintech), followed by incubation with 60 μl of protein G agarose beads for 6 h. After washing and reversing cross-linking, the input and immunoprecipitated DNA was purified and analyzed by real-time PCR using primers specifically targeting the HIV-1 LTR.

### Flow cytometry.

J-Lat A2 and J-Lat 10.6 cells (5 × 10^5^ cells/well) were incubated with the indicated concentrations of compounds for 48 h in 48-well plates. After washing three times with PBS, GFP expression was analyzed with a BD FACSCanto II flow cytometer (San Jose, CA, USA). Data were analyzed using FlowJo software (TreeStar, San Carlos, CA, USA), and the percentage of GFP-positive cells within the entire population was used as a measure of HIV-1 reactivation.

### Cell viability.

TZM-bl cells (1 × 10^4^ cells/well) transfected with UHRF1 siRNA or off-target control siRNA were seeded in a 12-well plate and incubated for 48 h. Cells were washed with PBS twice and stained with fixable viability dye eFluor 780 (catalogue no. 65-0865-14; Thermo Fisher Scientific). Cell viability was evaluated with a BD FACSCanto II flow cytometer (San Jose, CA, USA). Data were analyzed using FlowJo software (TreeStar, San Carlos, CA, USA).

### Establishment of a CD4^+^ T cell model with latent HIV-1 infection.

Nanfang Hospital of Guangzhou approved this study, and the experiment was performed in accordance with relevant guidelines and regulations. All donors gave written informed consent. Latently infected CD4^+^ T cell experiments were performed as described previously by Lewin’s group (27), with minor modifications. Briefly, PBMCs from a single healthy donor were isolated from buffy coats, and primary CD4^+^ T cells were isolated from the PBMCs by using an EasySep negative selection kit (StemCell Technologies, Inc., Vancouver, BC, Canada) according to the manufacturer’s instructions. After purification, the cells were maintained in complete RPMI 1640 medium supplemented with 29 nM CCL19 (R&D Systems) for 3 days. The cells were washed and infected with the HIV-1_NL4-3_ virus (100 ng of p24 viral equivalents per million cells) by spinoculation for 2 h at 1,000 × *g*. After infection, the cells were washed three times and cultured in the presence of interleukin-2 (IL-2; 10 IU/ml; Roche) for 6 days. Next, resting CD4^+^ T cells were isolated with a positive CD4^+^ T-cell selection kit (StemCell Technologies, Inc., Vancouver, BC, Canada) using a cocktail of antibodies against CD25-PE (clone M-A251; BD Pharmingen), CD69-PE (BD Pharmingen, clone FN50), and HLA-DR-PE (clone G46-6; BD Pharmingen).

### Statistical analyses.

Experimental data are presented as the means ± standard deviations (SD) from at least three independent experiments. Statistical analyses were conducted with GraphPad Prism 5.0 (GraphPad Software, San Diego, CA, USA). Comparisons between two groups were done using unpaired Student's *t* test, and one-way analysis of variance (ANOVA) followed by Dunnett’s multiple-comparison *post hoc* test was used to detect differences between the groups. A *P *value of <0.05 was used to indicate significance: *, *P < *0.05; **, *P* < 0.01; ***, *P *< 0.001.

10.1128/mBio.01625-21.4 TABLE S1 Primary antibodies for Western blotting. Download Table S1, DOC file, 0.04 MB. Copyright © 2021 Liang et al. 2021 Liang et al. https://creativecommons.org/licenses/by/4.0/ This content is distributed under the terms of the Creative Commons Attribution 4.0 International license.

10.1128/mBio.01625-21.5 TABLE S2 Chemicals and other reagents. Download Table S2, DOCX file, 0.01 MB. Copyright © 2021 Liang et al. 2021 Liang et al. https://creativecommons.org/licenses/by/4.0/ This content is distributed under the terms of the Creative Commons Attribution 4.0 International license.
