# Supplementary figures and images for: UHRF1 Suppresses HIV-1 Transcription and Promotes HIV-1 Latency by Competing with p-TEFb for Ubiquitination-Proteasomal Degradation of Tat
Source: mBio. 2021 Aug 31;12(4):e01625-21. doi: 10.1128/mBio.01625-21 (PMC8406157; doi:10.1128/mBio.01625-21)

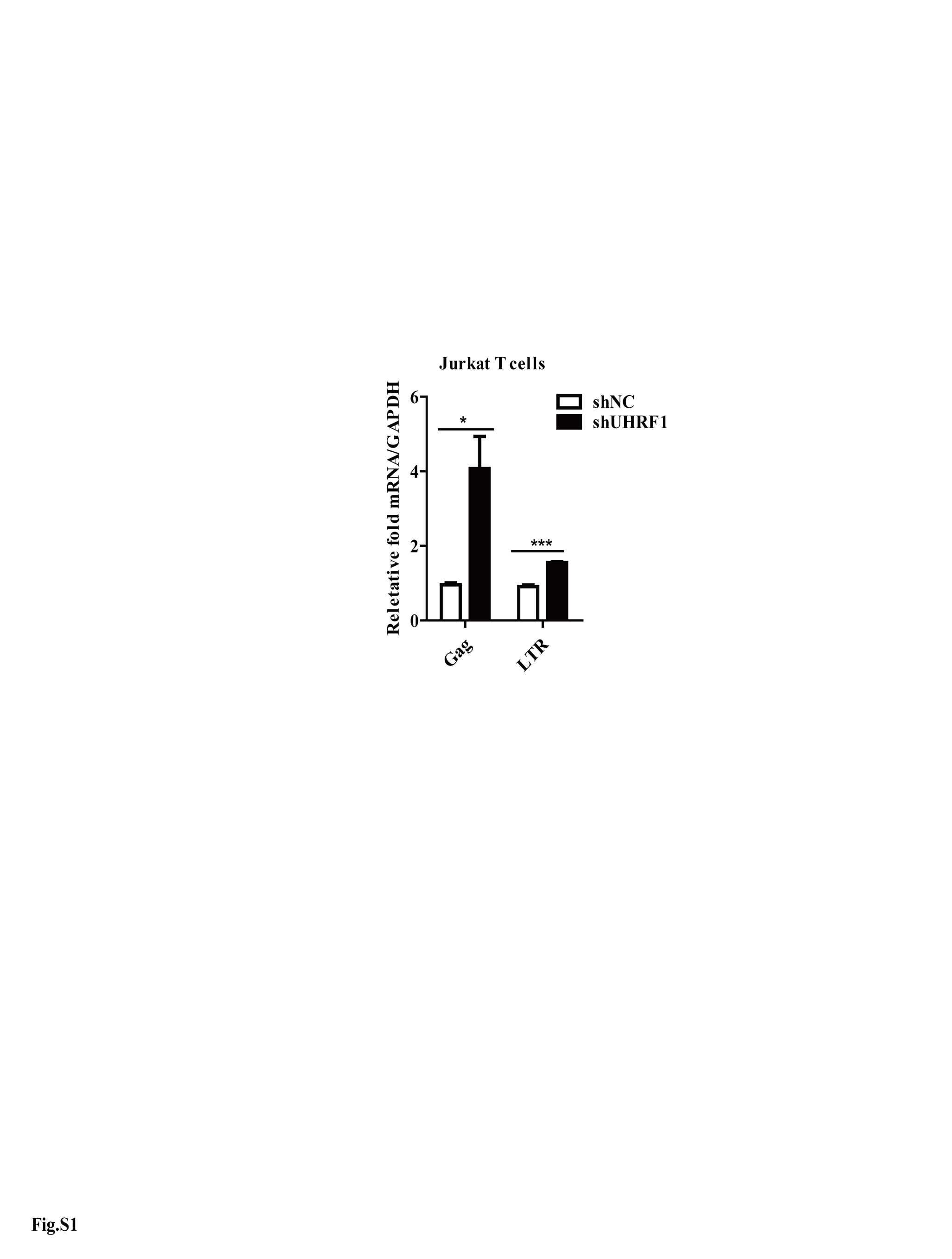

Supplement: FIG S1 [file mbio.01625-21-sf001.tif]

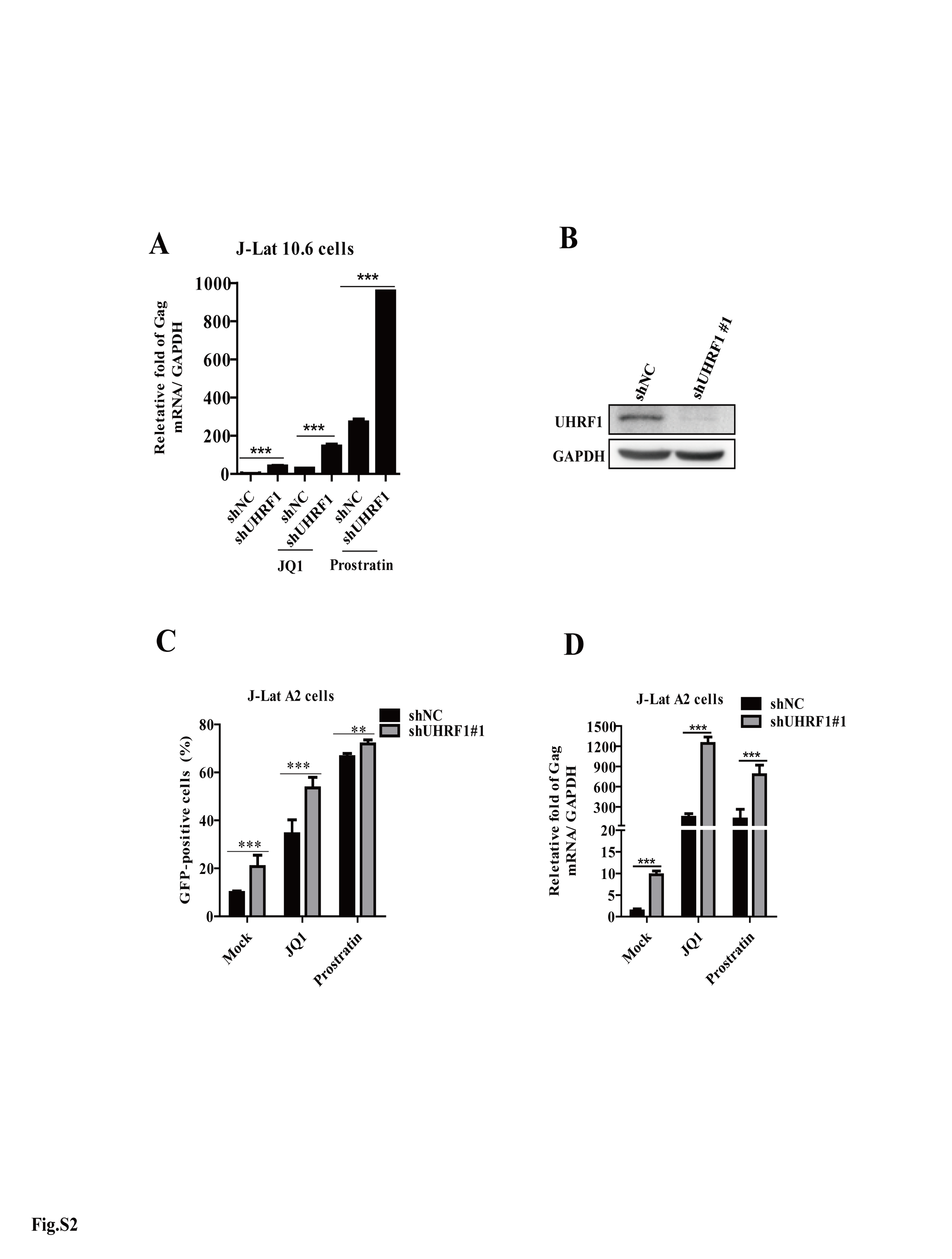

Supplement: FIG S2 [file mbio.01625-21-sf002.tif]

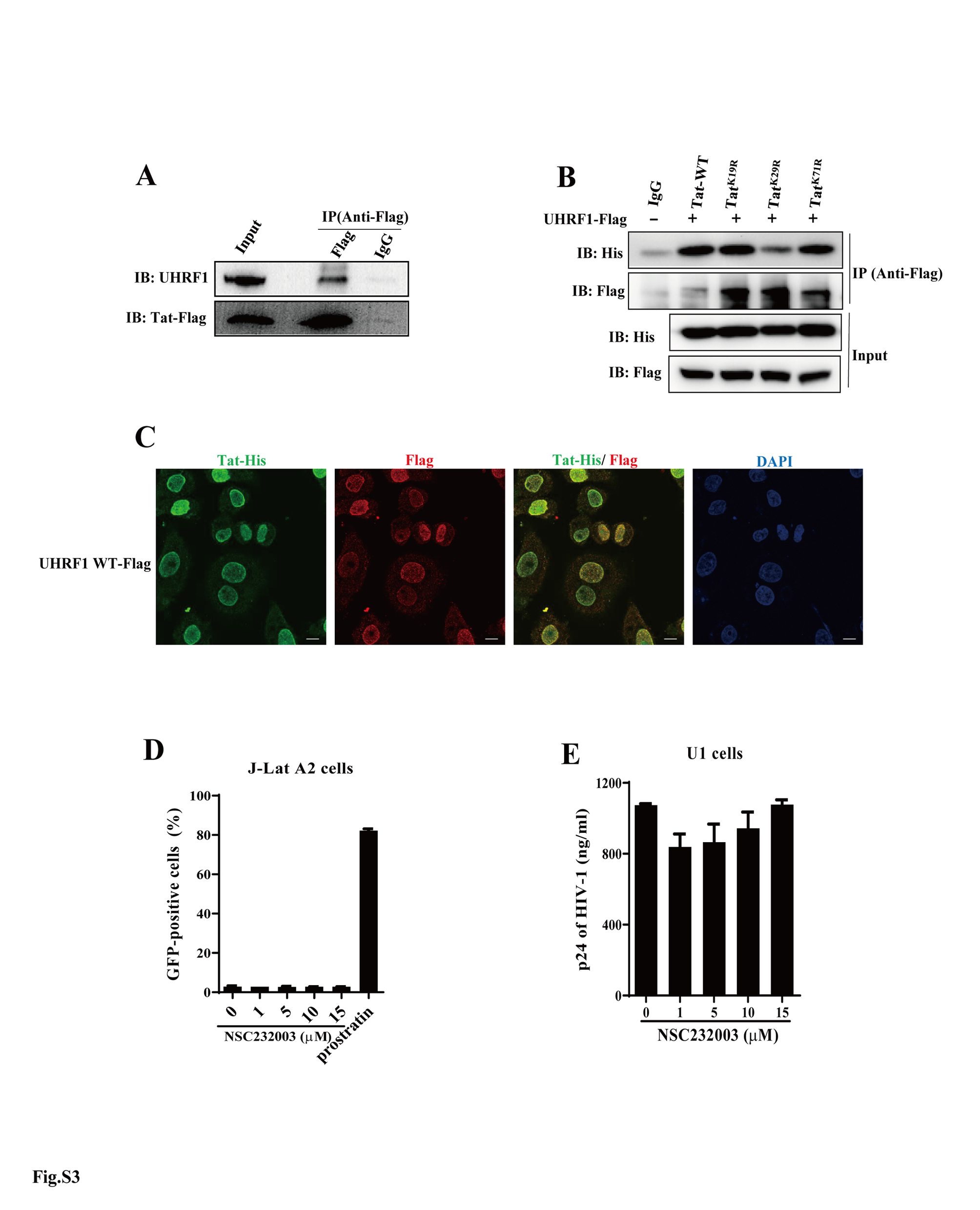

Supplement: FIG S3 [file mbio.01625-21-sf003.tif]
