# Supplementary material for: UHRF1 Suppresses HIV-1 Transcription and Promotes HIV-1 Latency by Competing with p-TEFb for Ubiquitination-Proteasomal Degradation of Tat
Source: mBio. 2021 Aug 31;12(4):e01625-21. doi: 10.1128/mBio.01625-21 (PMC8406157; doi:10.1128/mBio.01625-21)
Supplement: TABLE S1 [file mbio.01625-21-st001.doc]

**TABLE S1. Primary antibodies for Western blot.**

| **Target** | **Vendor** | **Cat. No** |
| --- | --- | --- |
| UHRF1 | Santa Crus | sc-398953 |
| GAPDH | Cell Signaling Technology | 5174 |
| β-actin | Cell Signaling Technology | 4970 |
| CDK9 | Santa Crus | sc-376646 |
| p-CDK9 (Thr186) | Cell Signaling Technology | 2549 |
| RNA Pol II | Cell Signaling Technology | 2629 |
| P-S2-RNA Pol II | Cell Signaling Technology | 13499 |
| Cyclin T1 | Cell Signaling Technology/ Santa Crus | 81464/sc-271348 |
| Flag-Tag | Sigma | F1804 |
| HIV-1 p24 | Produced in our lab |  |
| H3K9me3 | Cell Signaling Technology | 9733 |
| HIV-1 Tat | Santa Crus | Sc-65912 |
| [His-Tag](http://www.casmart.com.cn/product-details/page/550/29506310) | Proteintech | 66005-1-lg |
| [Mouse/Rabbit IgG](http://www.casmart.com.cn/product-details/page/550/117254242) | Proteintech | B900620/66467-1-Ig |
