# Supplementary material for: UHRF1 Suppresses HIV-1 Transcription and Promotes HIV-1 Latency by Competing with p-TEFb for Ubiquitination-Proteasomal Degradation of Tat
Source: mBio. 2021 Aug 31;12(4):e01625-21. doi: 10.1128/mBio.01625-21 (PMC8406157; doi:10.1128/mBio.01625-21)
Supplement: TABLE S2 [file mbio.01625-21-st002.docx]

**TABLE S2. Chemicals and other reagents**

| **Reagent** | | **Vendor** | **Cat. No** |
| --- | --- | --- | --- |
| JQ1 | | MedChemExpress | 202592-23-2 |
| prostratin | | Sigma | 60857-08-1 |
| NSC232003 | | MedChemExpress | 1905453-18-0 |
| MG-132 | | Selleckchem | S2619 |
| chloroquine | | Selleckchem | S6999 |
| Bafilomycin | | Selleckchem | S1413 |
| Cycloheximide | | Selleckchem | S7418 |
| TNF-α | | Sigma | 94948-59-1 |
| Lipofectamine 2000 | | Thermo Fisher Scientific | 11668019 |
| Chromatin  Immunoprecipitation (ChIP) assay kit | | Millipore | 17-295 |
| PolyJet^TM^ | | SignaGen | SL100688 |
| Luciferase Assay System | | Promega | E1501 |
| Total RNA Isolation Kit | | Foregene | RE-03113 |
|  |  | |  |
|  |  | |  |
|  |  | |  |
|  |  | |  |
